# Ascorbic acid induces global epigenetic reprogramming to promote meiotic maturation and developmental competence of porcine oocytes

**DOI:** 10.1038/s41598-018-24395-y

**Published:** 2018-04-17

**Authors:** Xiao-Xia Yu, Yun-Hua Liu, Xiao-Man Liu, Pei-Chao Wang, Shuai Liu, Jia-Kun Miao, Zhi-Qiang Du, Cai-Xia Yang

**Affiliations:** College of Animal Science and Technology, Northeast Agricultural University, Harbin, 150030 Heilongjiang China

## Abstract

L-ascorbic acid (Vitamin C) can enhance the meiotic maturation and developmental competence of porcine oocytes, but the underlying molecular mechanism remains obscure. Here we show the role of ascorbic acid in regulating epigenetic status of both nucleic acids and chromatin to promote oocyte maturation and development in pigs. Supplementation of 250 μM L-ascorbic acid 2-phosphate sesquimagnesium salt hydrate (AA2P) during *in vitro* maturation significantly enhanced the nuclear maturation (as indicated by higher rate of first polar body extrusion and increased *Bmp15* mRNA level), reduced level of reactive oxygen species, and promoted developmental potency (higher cleavage and blastocyst rates of parthenotes, and decreased *Bax* and *Caspase3* mRNA levels in blastocysts) of pig oocytes. AA2P treatment caused methylation erasure in mature oocytes on nucleic acids (5-methylcytosine (5 mC) and *N*^*6*^-methyladenosine (m^6^A)) and histones (Histone H3 trimethylations at lysines 27, H3K27me3), but establishment of histone H3 trimethylations at lysines 4 (H3K4me3) and 36 (H3K36me3). During the global methylation reprogramming process, levels of TET2 (mRNA and protein) and *Dnmt3b* (mRNA) were significantly elevated, but simultaneously DNMT3A (mRNA and protein), and also *Hif-1α*, *Hif-2*α, *Tet3*, *Mettl14*, *Kdm5b* and *Eed* (mRNA) were significantly inhibited. Our findings support that ascorbic acid can reprogram the methylation status of not only DNA and histone, but also RNA, to improve pig oocyte maturation and developmental competence.

## Introduction

L-ascorbic acid (Vitamin C), a water-soluble antioxidant and electron donor, can be synthesized in the liver of many species, except for guinea pigs, human and other primates^1^. Ascorbic acid can be actively transported into cells to reach a high concentration up to 1~10 mM^1,2^, by the high affinity sodium-dependent vitamin C transporters 1 and 2 (SVCT1 and SVCT2)^3^. Accumulating evidences demonstrate that ascorbic acid plays an important role in multiple biological processes^4,5^ via its reductive form (ascorbate), to reduce free radicals and reactive oxygen species by serving as powerful antioxidant (non-enzymatic function), and as an essential cofactor to modulate the family of ferrous ion- and 2-oxoglutarate (Fe^2+^ and 2-OG)-dependent dioxygenases (enzymatic function)^6^. The dioxygenase enzyme family consists of several subgroups, exists widely in nature, and catalyzes epigenetic demethylation and hydroxylation reactions, to affect multiple biological processes, such as collagen biosynthesis, hypoxic sensing, lipid metabolism^7^ and pluripotency^8^. Subgroup I, the ten-eleven-translocation 1–3 (Tet1–3) enzymes, is known to catalyze the DNA demethylation by converting 5-methylcytosine (5 mC) to 5-hydroxymethylcytosine (5 hmC)^9^. Subgroup II, the AlkB dioxygenases, includes fat mass and obesity-associated (Fto) and AlkB homologue 5 (Alkbh5) genes, to remove the methyl group from *N*^*6*^-methyladenosine (m^6^A) in DNA or RNA^10,11^. Subgroup III, the Jumonji C (JmjC) domain containing histone lysine demethylases (JmjC-KDMs), erases the methyl group on lysine residues of histones^12^. Subgroup IV, the hypoxia-inducible factor (HIF) hydroxylases, catalyzes hydroxylation on specific proline and asparagine residues of the transcription factor HIF-1α^13^. Studies confirmed that ascorbic acid regulates Tet, JmjC domain containing enzymes and HIF hydroxylases, to modulate dynamically the epigenetic status of DNA/histone methylation and HIF-1α activity^6,9,12–14^. However, whether and how ascorbic acid act via AlkB dioxygenases to effect m^6^A modification are largely unknown.

Mammalian oocyte development is coordinated by a complex molecular network, and dynamic epigenetic methylation regulation on DNA and histones is crucial for both oocyte meiosis^15,16^ and early embryo development^17–19^. Histone H3 trimethylations at lysines 4 (H3K4me3) and 36 (H3K36me3) are associated with active chromatin status, and trimethylations at lysines 9 (H3K9me3) and 27 (H3K27me3) represent repressive status^20,21^, which have critical epigenetic roles in regulating oogenesis and embryogenesis^22^. It is well known that the *in vitro* culture and maturation system of mammalian oocytes is often far from optimization, and as a result, large amount of reactive oxygen species (ROS) will usually be induced. Normally, ROS can be neutralized by the antioxidant defense system. However, when the net ROS level is above the physiological threshold, oxidative stress will occur, and thereby decrease oocyte quality and hinder subsequent embryonic development^23^. Recently, RNA methylation was also found to play an important role in oocyte meiosis in *Xenopus laevis*^24^, and maternal-to-zygote transition of early embryos in zebrafish^25^.

Furthermore, ascorbic acid treatment during *in vitro* maturation can enhance the nuclear maturation of porcine oocytes devoid of cumulus cells^26^, increase intracellular glutathione (GSH) level, reduce ROS level of porcine oocytes enclosed with cumulus cells^27^, and improve developmental competence of porcine oocytes after parthenogenetic activation^26,27^, *in vitro* fertilization^28^ and somatic cell nuclear transfer^27^. Supplementation of ascorbic acid during embryo culture can also improve blastocyst development of porcine hand-cloned embryos^29^ and mouse embryos made by somatic cell nuclear transfer^30^. However, the underlying molecular mechanism remains unknown.

In the present study, we aimed to understand how ascorbic acid improves the maturation and developmental competence of porcine oocytes enclosed with cumulus cells, with a special focus on epigenetic regulation. Here we showed that through regulating global epigenetic modifications at DNA, RNA and histone levels, supplementation of ascorbic acid during *in vitro* maturation can benefit the meiotic maturation and subsequent development of pig oocytes.

## Results

### AA2P treatment promotes first polar body extrusion of pig oocytes

**Figure 1:**
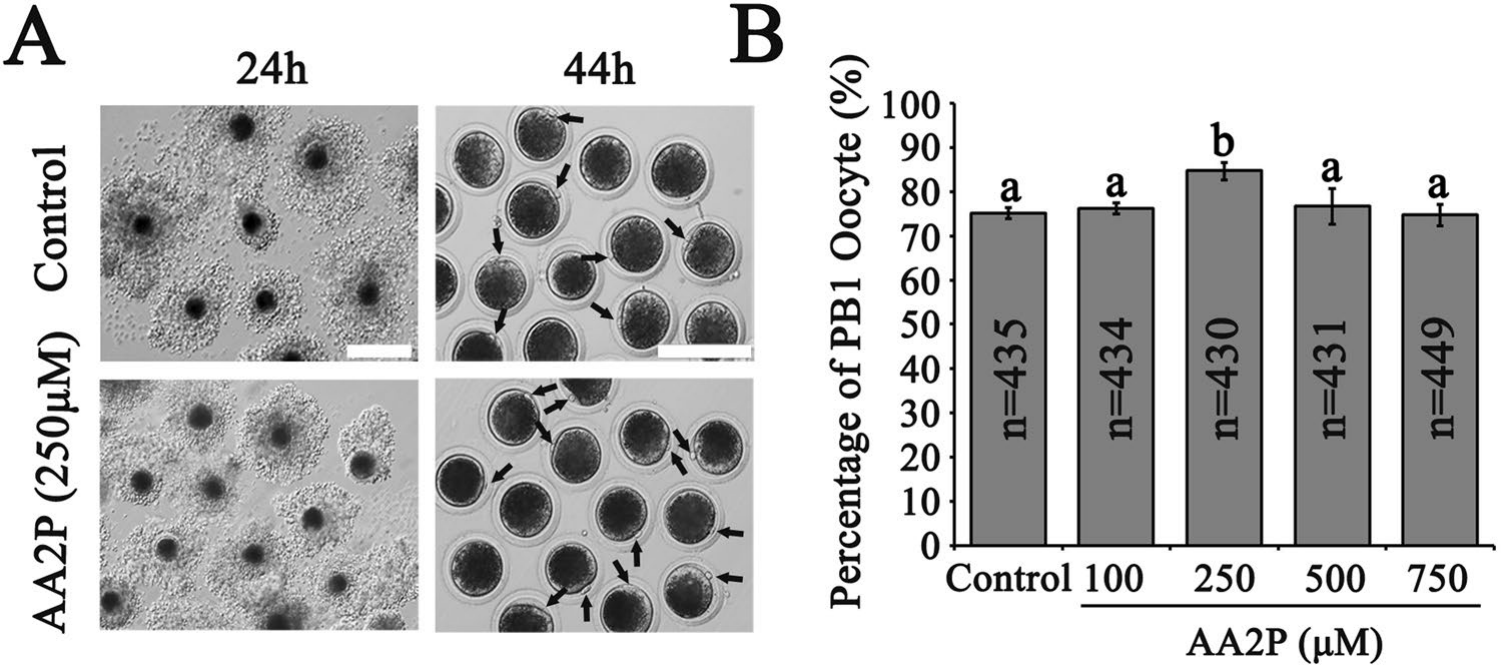
AA2P promotes the nuclear maturation of pig oocytes. (**A**) Representative images of porcine COCs *in vitro* matured for 24 h (left column) and denuded oocytes from *in vitro* matured COCs for 44 h (right column). Images in the left column showed cumulus extension with no differences among control and 250 µM AA2P treated groups, and ones in right column showed the morphology of oocytes after 250 µM AA2P treated for 44 h during IVM. Scale bar: 200 µm. (**B**) 250 µM AA2P treatment significantly increased the polar body 1 (PB1) extrusion rate of porcine oocytes. Different lowercase letters a and b indicate significant differences at *P* < 0.05 level.

Addition of L-ascorbic acid 2-phosphate sesquimagnesium salt hydrate (AA2P) into the *in vitro* maturation (IVM) system to culture pig cumulus-oocyte complexes (COCs) for 44 h showed that the rate of the first polar body (PB1) extrusion was significantly higher in 250 µM AA2P group (84.8%, n = 430) in comparison to the control (vs. 75.2%, n = 435; *P* < 0.01), 100 µM (vs. 76.3%, n = 434; *P* < 0.01), 500 µM (vs. 76.7%, n = 431; *P* < 0.05) and 750 µM (vs. 74.7%, n = 449; *P* < 0.01) groups (Fig. 1A and B). No significant differences existed between the other groups. Since both Svct1 and Svct2 are transporters of ascorbic acid, we then detected SVCT1 and SVCT2 transcript levels in porcine mature oocytes using RT-qPCR, where *Svct2* was relatively highly expressed than *Svct1* (Supplementary Table S1). However, 250 µM AA2P treatment did not significantly change mRNA levels of *Svct1* and *Svct2* (Supplementary Fig. S1). Therefore, treatment of porcine COCs using ascorbic acid at optimal concentration during IVM could promote the nuclear maturation of oocytes.

### AA2P suppresses the ROS level and increases Bmp15 mRNA level

**Figure 2:**
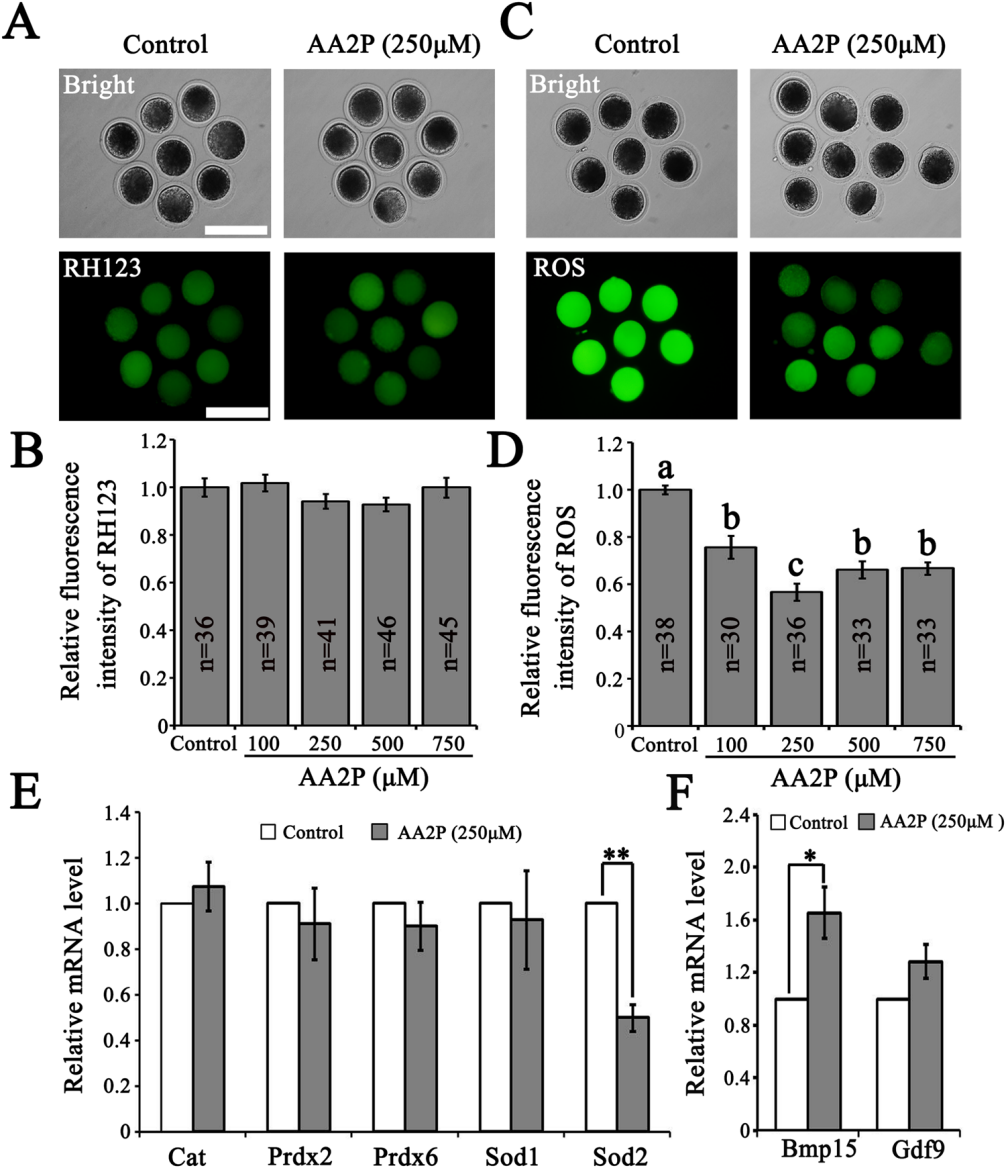
AA2P improves the cytoplasmic quality of pig oocytes. (**A**,**C**) The representative images of RH123 and ROS staining in oocytes from control and 250 µM AA2P treated groups. Scale bar: 200 µm. (**B**,**D**) The fluorescence values of RH123 and ROS levels in oocytes from control and AA2P treated groups. Different lowercase letters a, b and c indicate significant differences at *P* < 0.05 level. (**E**,**F**) RT-qPCR analysis on oxidative stress-related genes (*Cat*, *Prdx2*, *Prdx6*, *Sod1* and *Sod2*) and oocyte secreted factors (*Bmp15* and *Gdf9*). *indicates significant difference at *P* < 0.05 level, **P < 0.01 level.

To further investigate the effect of AA2P on porcine oocyte quality, we examined intracellular ROS production, mitochondrial membrane potential (ΔΨm) level and mRNA levels of oocyte secreted factors in MII oocytes from the control and AA2P treatment groups. AA2P did not affect the mitochondrial ΔΨm levels regardless of treatment concentrations (control vs. 100 µM vs. 250 µM vs. 500 µM vs. 750 µM: 1.00 vs. 1.02 vs. 0.94 vs. 0.93 vs. 1.00; *P* > 0.05; Fig. 2A and B). However, as compared to the control group, ROS levels were significantly decreased by AA2P treatments at all concentrations (control vs. 100 µM vs. 250 µM vs. 500 µM vs. 750 µM: 1.00 vs. 0.76 vs. 0.57 vs. 0.66 vs. 0.67; *P* < 0.001); specifically, 250 µM AA2P treatment even decreased ROS to a extremely lower level as compared to other AA2P groups (*P* < 0.05; Fig. 2C and D). Further RT-qPCR analyses on oxidative stress-related genes (*Cat*, *Prdx2*, *Prdx6*, *Sod1* and *Sod2*) and oocyte secreted factors (*Bmp15* and *Gdf9*) showed that AA2P treatment (250 µM) significantly down-regulated *Sod2* (0.50 vs. 1.00 of control; *P* < 0.01; Fig. 2E) and up-regulated *Bmp15* (1.66 vs. 1.00 of control; *P* < 0.05; Fig. 2F) levels. Our results indicate that ascorbic acid treatment could suppress ROS production, increase *Bmp15* mRNA level and thus improve cytoplasmic quality of porcine oocytes.

### AA2P elevates developmental potency of mature oocytes

**Figure 3:**
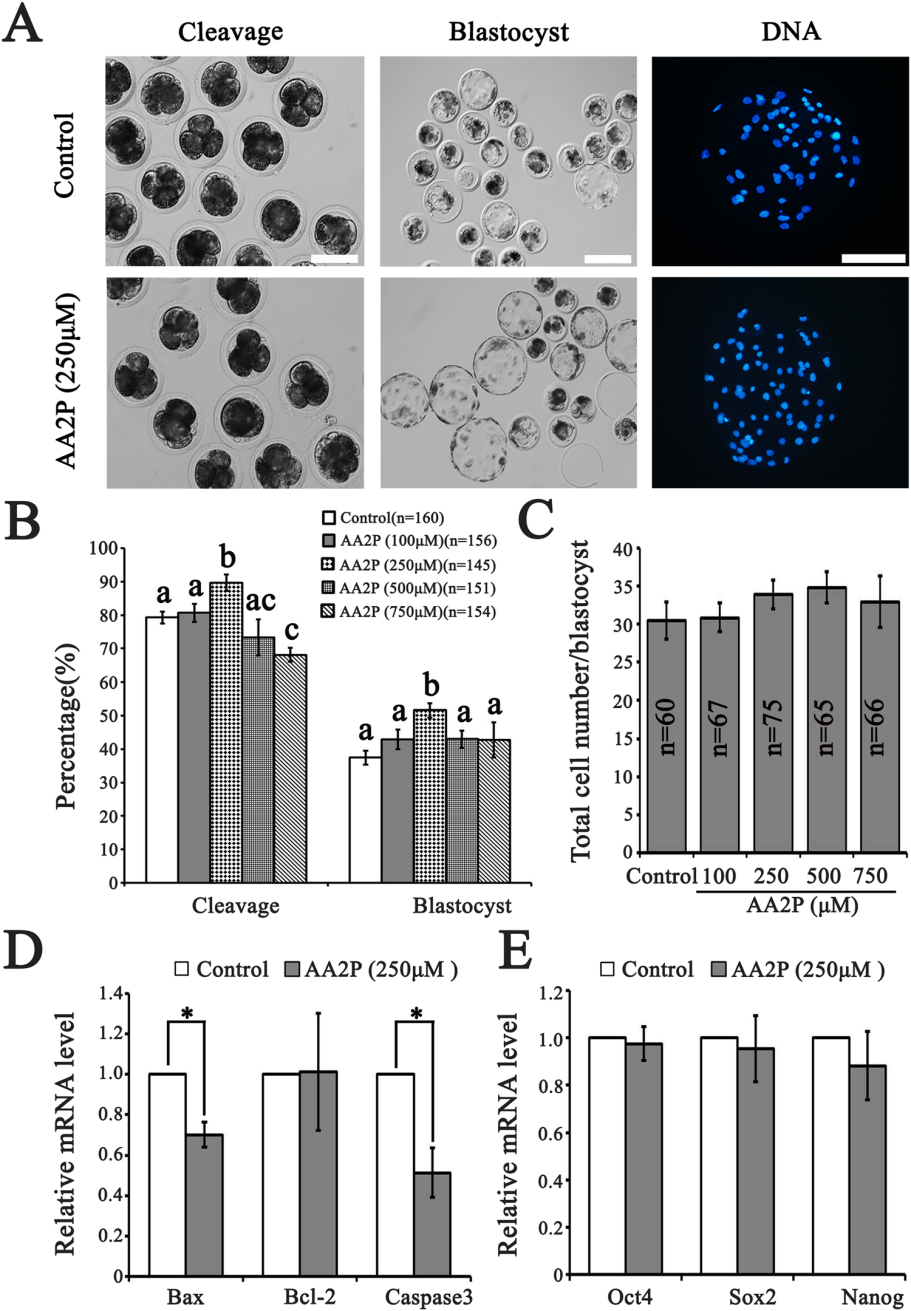
AA2P enhances the subsequent development of pig oocytes. (**A**) The representative images of parthenotes cleaved, developed to blastocyst and stained to show total cell number per blastocyst. Scale bar: 200 µm. (**B**) Cleavage and blastocyst rates of parthenotes from control and AA2P treated groups. Different lowercase letters a, b and c indicate significant differences at *P* < 0.05 level. (**C**) The average total cell number per blastocyst from control and AA2P treated groups. (**D**) Relatvie mRNA levels of apoptosis-related genes (*Bax, Bcl-2* and *Caspase3*). *Indicates significant difference at *P* < 0.05 level. (**E**) Relative mRNA levels of pluripotency genes (*Oct4*, *Sox2* and *Nanog*).

To investigate whether ascorbic acid treatment during IVM affects subsequent development of porcine mature oocytes, we monitored the cleavage, formation and average cell number of blastocysts by parthenogenetically activating mature oocytes (Fig. 3A). The cleavage rate of parthenotes was significantly higher in 250 µM AA2P group (89.9%, n = 145) than the control (vs. 79.3%, n = 160; *P* < 0.01), 100 µM (vs. 80.7%, n = 156; *P* < 0.05), 500 µM (vs. 73.3%, n = 151; *P* < 0.01) and 750 µM (vs. 68.1%, n = 154; *P* < 0.001) groups. Moreover, the cleavage rate in 750 µM AA2P group was significantly lower than the control (*P* < 0.05), 100 µM (*P* < 0.01) AA2P groups (Fig. 3B). Till day 7, blastocyst rate of parthenotes significantly increased in the 250 µM AA2P group (51.6%), relative to the control (vs. 37.5%; *P* < 0.01), 100 µM (vs. 43.0%; *P* < 0.05), 500 µM (vs. 43.0%; *P* < 0.05) and 750 µM (vs. 42.8%; *P* < 0.05) AA2P groups (Fig. 3B). However, AA2P treatments did not affect the average total cell number per blastocyst, no matter what concentration (Fig. 3C).

We further detected the mRNA abundance of selected genes related to apoptosis (*Bax*, *Bcl-2* and *Caspase3*) and pluripotency (*Oct4*, *Sox2* and *Nanog*) in blastocysts, and found *Bax* (0.70 vs. 1.00 of control) and *Caspase3* (0.51 vs. 1.00 of control) to be significantly down-regulated (*P* < 0.05), whereas *Bcl-2* and pluripotency genes were unchanged in the 250 µM AA2P group as compared to the control group (*P* > 0.05; Fig. 3D and E). Our results clearly demonstrate that ascorbic acid treatment elevated the competence of porcine oocytes to further develop to blastocyst stage, partially through inhibiting apoptosis during early embryogenesis.

### AA2P decreases DNA 5 mC methylation

**Figure 4:**
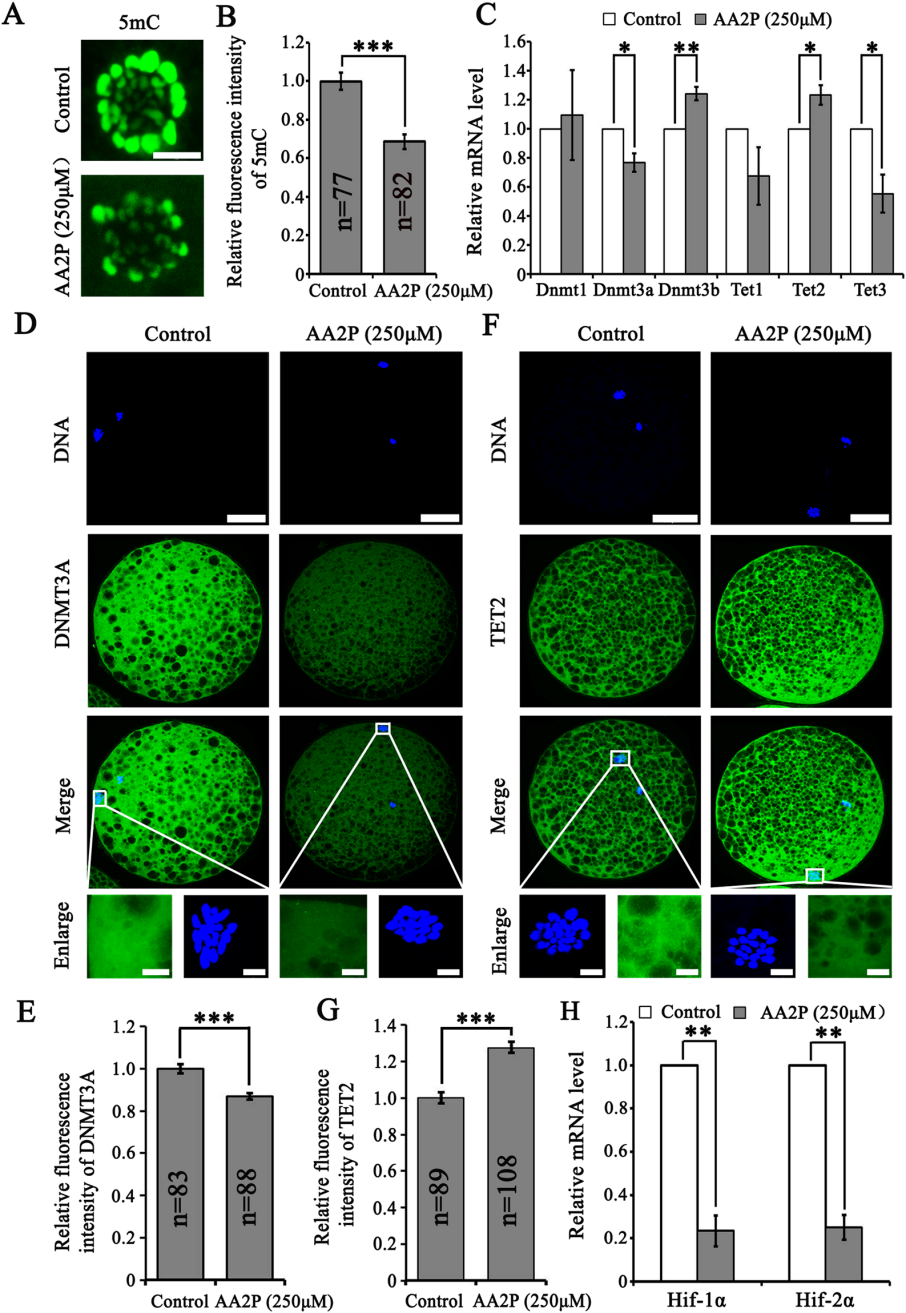
AA2P decreases 5 mC levels in pig oocytes. (**A**) Immunofluorescence staining of 5 mC in mature oocytes from control and 250 µM AA2P treated groups. (**B**) Quantitative analysis of 5 mC levels in mature oocytes from control and 250 µM AA2P treated groups. (**C**) Relative mRNA levels of 5 mC-related writers (*Dnmt1*, *Dnmt3a*, *Dnmt3b*) and erasers (*Tet1*, *Tet2* and *Tet3*) as detected by RT-qPCR. (**D**,**E**) Immunofluorescence staining and signal values of DNMT3A. (**F**,**G**) Immunofluorescence staining and signal values for TET2. (**H**) RT-qPCR analysis of *Hif-1α* and *Hif-2α*. Scale bar: 50 µm for first three rows in D and F, 5 µm in A and for final rows in D and F. *Indicates significant difference at *P* < 0.05 level, **P < 0.01 level and ***P < 0.001 level.

Ascorbic acid regulates TET family enzyme activity, and affects the conversion of 5 mC to 5 hmC. Therefore, we examined the global changes of 5 mC level and its related enzymes in porcine oocytes after AA2P treatment (250 µM). Immunofluorescence analysis showed that the whole genome 5 mC levels of mature oocytes after IVM were significantly decreased by AA2P treatment (1.00 vs. 0.69; *P* < 0.001; Fig. 4A and B). Transcript levels of 5 mC-related writers (*Dnmt1*, *Dnmt3a* and *Dnmt3b*) and erasers (*Tet1*, *Tet2* and *Tet3*) detected by RT-qPCR showed that AA2P treatment significantly up-regulated *Dnmt3b* (1.24 vs. 1.00 of control; *P* < 0.01) and *Tet2* (1.23 vs. 1.00 of control; *P* < 0.05), whereas significantly down-regulated *Dnmt3a* (0.77 vs. 1.00 of control; *P* < 0.05) and *Tet3* (0.68 vs. 1.00 of control; *P* < 0.05) (Fig. 4C). Further immunofluorescence staining confirmed the significantly decreased protein level of DNMT3A (0.87 vs. 1.00 of control; Fig. 4D and E) and increased level of TET2 (1.28 vs. 1.00 of control; *P* < 0.001; Fig. 4F and G) induced by AA2P, consistent with their gene expression changes. Our results indicate that ascorbic acid can reset DNA methylation writers and erasers, to decrease global 5 mC level.

Since in human melanoma cells ascorbic acid could suppress HIF-1α level and activity^31^, and knockdown of HIF-1α increased TET2 mRNA and protein expression^32^, as well as HIF-1α could transactivate DNMT3a in leukemia cells^33^, we examined AA2P’s effects on *Hif-1α* and *Hif-2α* in mature oocytes. Our results showed that AA2P significantly down-regulated *Hif-1α* (0.24 vs. 1.00 of control; *P* < 0.01) and *Hif-2α* (0.25 vs. 1.00 of control; *P* < 0.01) transcript levels (Fig. 4H). These data suggest that ascorbic acid could lower DNA 5 mC methylation level possibly via HIF-1α induced increase of TET2 and decrease of DNMT3A.

### AA2P reduces global level of ***N***^***6***^-methyladenosine modification

**Figure 5:**
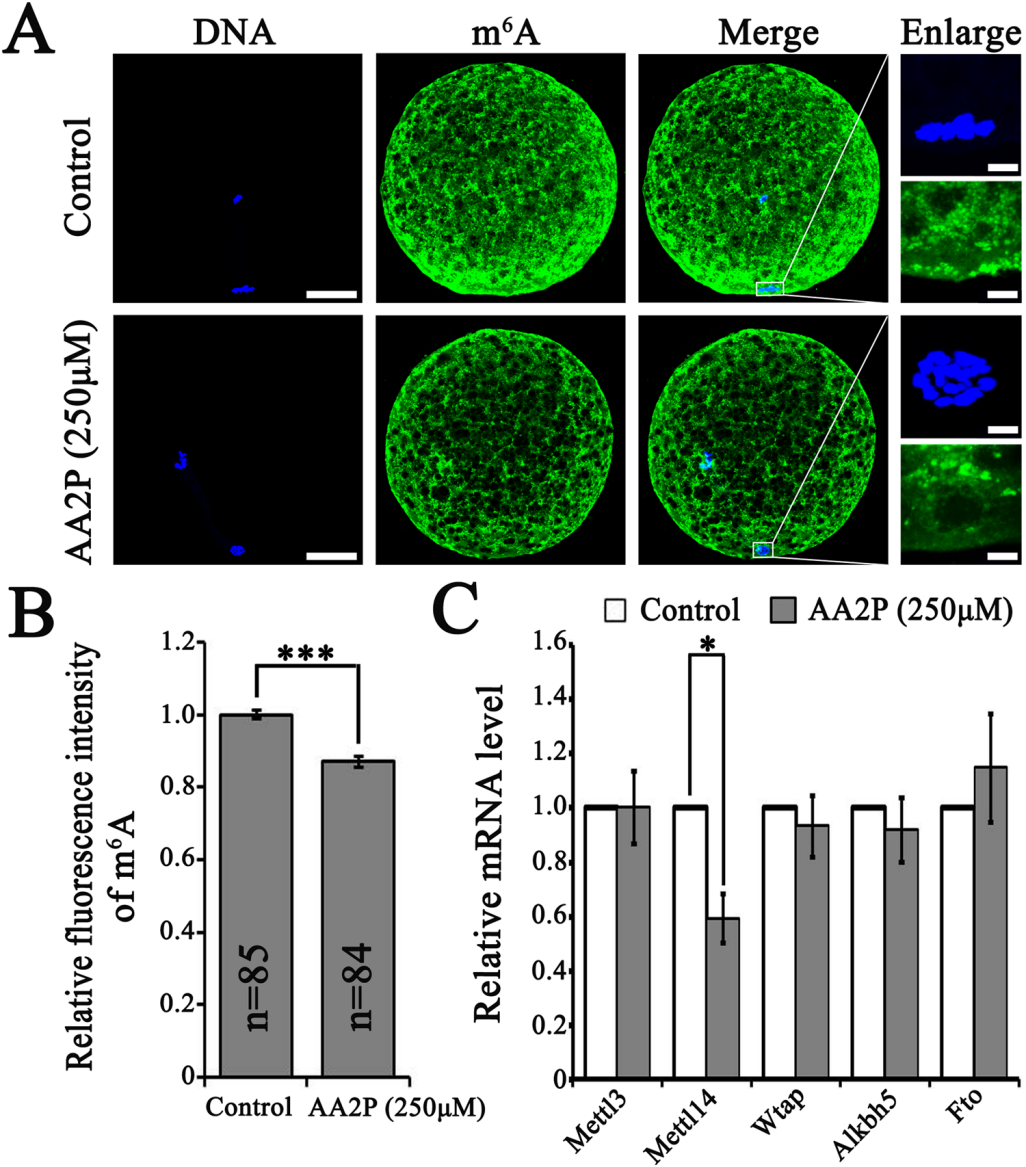
AA2P decreases global m^6^A level in pig oocytes. (**A**) Immunofluorescence staining of m^6^A modification. Scale bar: 50 µm for first three columns and 5 µm for final column. (**B**) Quantitative analysis of m^6^A level in mature oocytes from control and 250 µM AA2P treated groups. (**C**) Relative mRNA levels of m^6^A related genes (*Mettl3*, *Mettl14*, *Wtap*, *Alkbh5*, *Fto*). *Indicates significant difference at *P* < 0.05 level and ***P < 0.001 level.

To explore whether ascorbic acid affects global m^6^A level on both DNA and RNA, we performed m^6^A immunostaining on porcine mature oocytes using antibody specifically recognizing m^6^A. Fluorescent signal for m^6^A filled the whole area of ooplasm, with similar intensity for the chromosomal regions (Fig. 5A). After quantitative analysis of relative fluorescence intensity, global m^6^A level was most significantly decreased in oocytes treated by 250 µM AA2P, compared to the control oocytes (1.00 vs. 0.87; *P* < 0.001; Fig. 5B). mRNA levels of m^6^A related genes (*Mettl3*, *Mettl14*, *Wtap*, *Alkbh5* and *Fto*) showed that *Mettl14* was significantly reduced by 250 µM AA2P treatment (0.59 vs. 1.00 of control; *P* < 0.05; Fig. 5C). Thus, ascorbic acid could down-regulate the gene expression of *Mettl14* methyltransferase, and reduce global m^6^A level.

### AA2P modifies histone H3 trimethylation pattern

**Figure 6:**
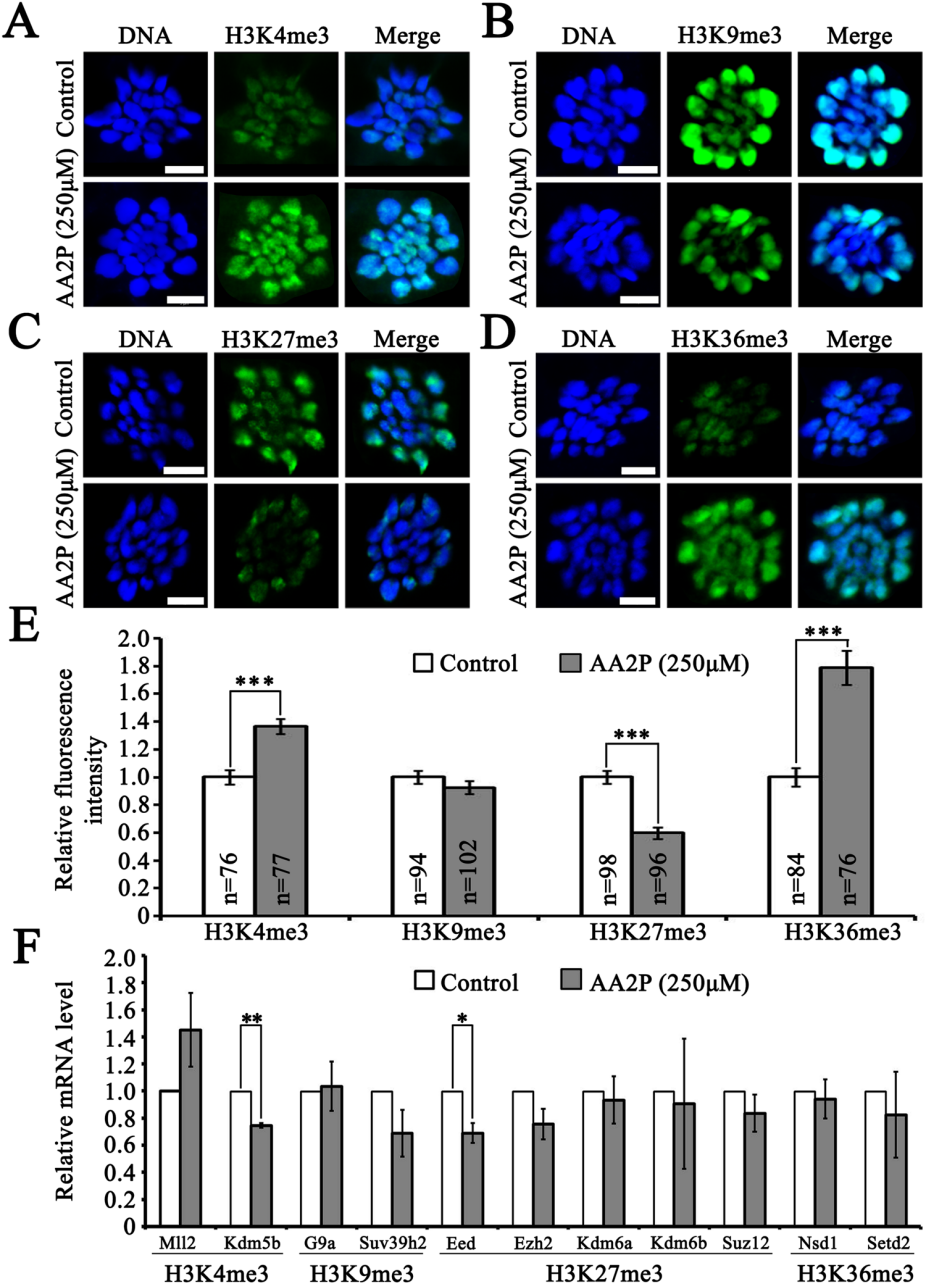
AA2P modifies histone methylation level of pig oocytes. (**A**–**D**) Immunofluorescence staining of H3K4me3, H3K9me3, H3K27me3 and H3K36me3 modifications. Scale bar: 5 µm. (**E**) Quantitative analysis of H3K4me3, H3K9me3, H3K27me3 and H3K36me3 levels. (**F**) Relative mRNA levels of genes related to histone methylation markers (*Mll2*, *Kdm5b*, *G9a*, *Suv39h2*, *Eed*, *Ezh2*, *Kdm6a*, *Kdm6b*, *Suz12*, *Nsd1* and *Setd2*). *Indicates significant difference at *P* < 0.05 level, **P < 0.01 level and ***P < 0.001 level.

We hypothesized that ascorbic acid might modify histone methylation modification, and thus account for better maturation and developmental competence of porcine oocytes. To test this hypothesis, the tri-methylation levels of histone H3 at four lysine positions (H3K4me3, H3K9me3, H3K27me3 and H3K36me3) in mature oocytes were compared using immunofluorescence staining. AA2P treatment (250 µM) significantly increased the H3K4me3 (1.36 vs. 1.00 of control) and H3K36me3 levels (1.79 vs. 1.00 of control) (*P* < 0.001; Fig. 6A,D,E), and significantly decreased the H3K27me3 level (0.60 vs. 1.00 of control; *P* < 0.001; Fig. 6C,E), but without any effect on H3K9me3 (0.92 vs. 1.00 of control; *P* > 0.05; Fig. 6B,E). We further characterized mRNA expression profiles of 11 genes related to these four markers (*Mll2*, *Kdm5b*, *G9a*, *Suv39h2*, *Eed*, *Ezh2*, *Kdm6a*, *Kdm6b*, *Suz12*, *Nsd1* and *Setd2*), and found significant decrease of *Kdm5b* (0.75 vs. 1.00 of control; *P* < 0.01) and *Eed* (0.69 vs. 1.00 of control; *P* < 0.05) in oocytes treated by 250 µM AA2P (Fig. 6F). Our results suggest that ascorbic acid could alter *Kdm5b* and *Eed* to reset histone H3 trimethylation, and switch from repressive chromatin status onto active status.

## Discussion

Ascorbic acid has been reported to play an important role in many biological processes, through acting as an electron donor, suppressing oxidative stress, and regulating epigenetic modifications at DNA and histone levels^3^. Here, we further confirmed ascorbic acid partially acts through epigenetic reprogramming to improve meiotic maturation and developmental competence of porcine oocytes.

Regarding the concentration of AA2P, one previous report studying the effect of ascorbic acid 2-O-alpha-glucoside on porcine oocytes was referred, which was set in a range of 0–750 μM and added into the maturation media of porcine COCs^28^. Consistent with previous reports on ascorbic acid or its derivative^26–28^, we confirmed AA2P at the optimal concentration (250 μM) could significantly promote meiotic maturation and developmental competence of porcine oocytes. Furthermore, for embryos constructed by the somatic cell nuclear transfer method, treatment of porcine COCs during IVM by ascorbic acid could also enhance their development^27,29^.

We demonstrated that ascorbic acid could attenuate ROS level caused by *in vitro* culture conditions and increase *Bmp15* mRNA level, thereby contributing to better porcine oocyte quality. Oxidative stress was reported to affect oocyte quality, and therefore influence fertilization and early embryo development^23^. Ascorbic acid was also shown to improve blastocyst development of porcine hand-cloned embryos mainly through the antioxidant pathway^29^. Evidences showed that oocyte-derived BMP15 could regulate the proliferation, apoptosis^34^ and extension^35^ of cumulus cells to affect quality and subsequent development of oocytes^36^. In porcine, 100 ng/ml BMP15 added individually into *in vitro* maturation medium of COCs did not change nuclear maturation rate of oocytes but did activate M-phase-promoting factor (MPF) and mitogen-activated protein kinase (MAPK) signals in oocytes^35^. As for the association of ascorbic acid with *Bmp15* mRNA level within oocytes, it was shown first here and awaits further investigation.

Considering that oxidative stress was one known factor to alter epigenetic status^37^, and that ascorbic acid could modify epigenome status^3,4^, we examined global epigenetic levels of DNA, RNA and histone in mature oocytes. Our results showed that ascorbic acid could induce the reduction of global 5 mC level in porcine mature oocytes. In human metastatic melanoma cells, deficiency of HIF-1α increased TET2 mRNA and protein expression, and ascorbic acid induced TET2 dependent 5 hmC formation^32^. Thus, one possible mechanism underlying 5 mC erasure in pig oocytes might be through ascorbic acid induced TET2 enhancement. Moreover, we also found ascorbic acid inhibits Dnmt3a mRNA and protein abundances in mature porcine oocytes. Previous studies reported that ascorbic acid could lower HIF-1α protein level and activity in human melanoma cells^31^, and HIF-1α could cause DNMT3A to be highly expressed in leukemia cells^33^. Combined with our observation that *Hif-1α* mRNA level was lowered down by ascorbic acid treatment, we proposed another possible mechanism that ascorbic acid might lower DNMT3A expression, and in turn reduce DNA 5 mC global methylation via down-regulating HIF-1α expression in porcine oocytes. How ascorbic acid increases *Dnmt3b* and decreases *Tet3* transcript levels in porcine oocytes awaits further investigation. In addition, ascorbic acid serves as a cofactor to activate TET activity in mouse embryonic fibroblasts, and induces TET to catalyze the hydroxylation of 5 mC to 5 hmC in DNA^38,39^ while oxidative stress could significantly increase the global 5 mC level via lowering TET activity^37^. We thus propose the third possibility that ascorbic acid acts as antioxidant to suppress ROS production and increase TET activity, therefore reduces global 5 mC level in porcine oocytes.

In eukaryotic cells, m^6^A modification is the most common and reversible modifications on both DNA and RNA molecules^40^. The abundant m^6^A modification in RNA has been proven to play vital roles in *Xenopus laevis* oocyte meiosis^24^ and zebrafish embryo maternal-to-zygotic transition^25^. In *Xenopus laevis*, mRNAs with lower m^6^A modification levels in germinal vesicle or metaphase II (MII) oocytes showed significant higher protein levels, which were mainly associated with cell cycle and translation pathways^24^. In the present study, we found ascorbic acid decreased global m^6^A level in MII oocytes possibly through inhibiting the expression of m^6^A methyltransferase *Mettl14*, which might affect the translation of genes important for cell cycle and developmental potency of pig oocytes. Exact identity of these genes associated with oocyte meiosis and early embryo development still needs further investigation. Our results suggest that through a novel epigenetic mechanism, ascorbic acid can regulate m^6^A status to affect oocyte maturation and developmental potency.

Ascorbic acid treatment significantly decreased H3K27me3, but increased H3K4me3 and H3K36me3 in MII oocytes. As H3K4me3 and H3K36me3 generally correlates with actively transcribed chromatin, whereas H3K9me3 and H3K27me3 associate with repressive chromatin^41^, our results suggest that ascorbic acid treatment could switch chromatin from repressive to active status. Despite the global transcription activity is limited in MII oocytes, the relatively active chromatin status might affect the later global transcription initiated during maternal-zygotic transition in early embryos, to benefit the subsequent embryo development. Supportive evidences also showed that H3K4me3^42^ and H3K27me3^43^ modifications are important to epigenetic maturation and developmental potential of mouse oocytes. As for how ascorbic acid changes the status of these histone lysine trimethylation markers, three possibilities might exist. First, ascorbic acid could directly induce changes of the expression or activity of JmjC-KDMs^44^. Second, since some JmjC-KDMs, including KDM5b, are known to be direct targets of HIF^41,45,46^, ascorbic acid could indirectly affect JmjC-KDMs through decreasing HIF level. Third, a significant decrease of ROS level after ascorbic acid treatment suggests that suppressing oxidative stress may be effective in inducing changes of global histone methylation. This is supported by a report that ascorbic acid could rescue the increased global level of H3K4me3 in immortalized human bronchial epithelial cells induced by oxidative stress, through acting as an antioxidant to deoxidize Fe (III) to Fe (II) and thereby modulating the activity of JmjC-KDMs^37^.

Taken together, our findings showed that 250 µM ascorbic acid treatment during IVM could improve pig oocyte meiotic maturation and developmental competence, via reprogramming the global methylation status of not only DNA and histone, but also RNA.

## Methods

### Ethics statement

All experimental materials and procedures taken in this study were reviewed and approved by the Animal Care Commission and Ethics Committee of Northeast Agricultural University P. R. China. All methods were performed in accordance with the approved guidelines and regulations.

### Chemicals and antibodies

Derivative of L-ascorbic acid, L-ascorbic acid 2-phosphate sesquimagnesium salt hydrate (AA2P; A8960; Sigma, St. Louis, MO, USA), was chosen to use in the present study^47,48^, considering that L-ascorbic acid is unstable under multifarious oxidative conditions, for example exposure to heat and light^49,50^. All other reagents used in the present study were purchased from Sigma, unless otherwise stated. Antibodies for immunofluorescence assays included rabbit anti-DNMT3A monoclonal antibody (Abcam, Shanghai, China), rabbit anti-TET2 polyclonal antibody (Abclonal, Nanjing, China), rabbit anti-5 mC polyclonal antibody (Abclonal), rabbit anti-m^6^A monoclonal antibody (Abcam), rabbit anti-H3K4me3, H3K9me3, H3K27me3 and H3K36me3 polyclonal antibodies (Abclonal), fluorescein isothiocyanate (FITC) conjugated goat anti-rabbit IgG (H + L) antibody (Transgen).

### Collection and ***in vitro*** maturation (IVM) of pig COCs

Porcine ovaries were picked from a local slaughterhouse and then shipped to the laboratory within 2 h in sterile physiological saline (0.9% sodium chloride) containing penicillin and streptomycin at 30–35 °C. Follicular fluids were aspirated from antral follicles (about 3–5 mm in diameter) using an 18-gauge needle attached to a 5 ml disposable syringe, and washed three or four times in HEPES-buffered Tyrode medium (3.2 mM KCl,114 mM NaCl, 2 mM CaCl_2_·2H_2_O, 0.34 mM Na_2_HPO_4_, 0.5 mM MgCl_2_, 10 mM Na Lactate, 10 mM HEPES, 0.2 mM Na Pyruvate, 12 mM Sorbitol, 2 mM NaHCO_3_, 0.1 mg/ml polyvinylalcohol, 1 μg/ml Gentamicin). Then COCs with more than three layers of cumulus cells and uniform ooplasm were picked and washed three times in maturation medium (TCM 199 medium (Gibco BRL, Grand Island, NY) supplemented with 0.1% PVA, 3.05 mM D-glucose, 0.91 mM sodium pyruvate, 1 µg/ml gentamicin, 0.57 mM cysteine, 0.5 µg/ml luteinizing hormone, 0.5 µg/ml follicle stimulating hormone, 10 ng/ml epidermal growth factor). Then about 50 COCs were transferred into one well of a 24-well plate with 500 μl maturation medium covered by mineral oil and then cultured in an incubator (39 °C, 5% CO_2_, and saturated humid air) for 44 h. After *in vitro* maturation, cumulus cells were kicked off from the oocytes via votexing in 0.1% hyaluronidase solution in HEPES-buffered Tyrode medium containing 0.01% PVA. The cumulus-free oocytes were fixed with 4% paraformaldehyde in the phosphate buffered saline (PBS) solution for 40 min and stained with Hoechst33342 (10 µg/ml) for 10 min. Stained oocytes were mounted onto slides to examine nuclear status under an inverted fluorescence microscope (Olympus, Tokyo, Japan). Oocyte with the presence of PB1 was considered to be mature. AA2P was added into maturation medium at the final concentration as desired (0, 100 µM, 250 µM, 500 µM, 750 µM)^28^, to observe its effect on oocyte maturation (PB1 rate, ROS level, mitochondrial membrane potential) and subsequent embryo development (cleavage rate, blastocyst rate and cell number per blastocyst). According to the data collected, we then assessed the AA2P dose effects, and chose the optimal concentration to further investigate the epigenetic changes and mRNA abundance of related genes.

### Parthenogenetic activation and embryo ***in vitro*** culture

The mature oocytes were washed using activation medium (0.28 M mannitol, 0.1 mM CaCl_2_·2H_2_O, 0.1 mM MgCl_2_, 1 mg/ml BSA, 0.5 mM HEPES) and stimulated using two direct pulses of 1.2 kV/cm for 30 μs on Electrocell Manipulator (BTX830, USA). Then, oocytes were incubated for 4 h in porcine zygote medium 3 (PZM-3) with 2.5 mM 6-dimethylaminopurine and 5 μg/mL cytochalasin B in the incubator (39 °C, 5% CO_2_ and saturated humid air). Activated embryos were cultured in PZM-3 covered with mineral oil in the incubator for 7 days and the cleavage and blastocyst rates were recorded at 48 h and 168 h post-activation. Cell number of blastocysts was counted after Hoechst33342 staining.

### Immunofluorescence staining and quantification

Denuded MII oocytes were fixed with 4% paraformaldehyde in PBS for 40 min at room temperature (RT), permeabilized using 1% Triton X-100 in PBS overnight at 4 °C. Then, samples were blocked in 1% bovine serum albumin (BSA) in PBS for 1 h, and incubated overnight at 4 °C with first antibodies. After washed with PBST (PBS plus 0.01% Triton X-100 and 0.1% Tween-20) three times (10 min each time), oocytes were incubated with second antibody (1:150 in blocking reagent) for 1 h at RT. Then, samples were stained with 10 μg/ml Hoechst33342 for 10 min in PBS after washed three times with PBST. Last, oocytes were mounted onto glass slides in ProLong Diamond Antifade Mountant reagent (Life Technologies, USA) and images were taken under a fluorescence microscope (Nikon 80i, Japan). Samples incubated without the primary or secondary antibodies were set as negative controls. Quantification of fluorescence intensity was performed using Image J software (version 1.48 V; NIH, Bethesda, MD, USA). For quantification of signal located in nuclear area, the average pixel from three different cytoplasm areas were used as background for normalization. When quantification of signal located in whole oocyte, the average pixel from five negative control oocytes (omitting first antibody) was set as background for normalization. The net signal intensity was generated using the average pixel intensity of sample oocyte to subtract the background. At least 75 oocytes in each group were analyzed. Representative images were taken using a Laica laser-scanning confocal microscope (Leica, Germany). For 5 mC immuno-detection, denuded MII oocytes were fixed for 40 min at RT in 4% paraformaldehyde, permeabilized using 0.5% Triton X-100 for 40 min at RT. Then, samples were treated with 4 N HCl for 50 min at 37 °C. After 4 washes in 0.05% Tween-20, oocytes were blocked for 1 h in PBS containing 2% BSA and incubated with a antibody against 5 mC (1:150) for 1 h at 37 °C. Several washes and 1 h incubation with a FITC-conjugated secondary antibody (1:150) later, oocytes were postfixed overnight at 4 °C in 4% paraformaldehyde. Thereafter, chromatin staining, sample mounting, image taking and fluorescence quantification were performed as described above.

### Measurement of reactive oxygen species (ROS) and mitochondrial membrane potential (ΔΨm)

The MII oocytes were sampled to determine intracellular ROS levels by methods described previously^51^. Briefly, oocytes were incubated in 10 µM 2′,7′-dichlorodihydrofluorescein diacetate in PBS at 37 °C for 30 min. After washed 3 times in PBS, samples were placed into 10 µl PBS droplets to take images under an inverted fluorescence microscopy (Olympus, Tokyo, Japan) using the same parameter settings. The fluorescence intensity of each oocyte was analyzed using Image J software and the average value for the control group was set as the reference.

The mitochondrial ΔΨm in denuded MII oocytes were assessed using the Rhodamine 123 (RH123) staining, according to a previous report^51^. Briefly, samples were incubated with 5 μg/ml RH123 for 30 min at 37 °C in dark. After washed with PBS, the fluorescent images were taken and analyzed in a similar way as mentioned for ROS level.

### Real-time PCR

60 MII oocytes or 20 blastocysts were used to extract total RNA using RNeasy Mini Kit (Qiagen, Germany) according to the manufacture’s instructions. RNase-free Dnase set (Qiagen) was used to remove genomic DNA. Total RNA was reverse transcribed using 20 μl reaction system (2 ul RT Buffer, 0.8 μl dNTP mix, 2 μl RT random primer, 1 μl multiscribe reverse transcriptase, 14.2 μl RNA within Rnase-free water) (ABI, Life technologies). Quantitative PCR was conducted in a 10 μl system including 5 μl fast-start universal SYBR green master mix, 0.3 μl primers, 1 μlcDNA, 3.7 μl RNase-free water) (Roche Molecular Systems) on the 7500 real-time PCR detection system (Applied Biosystems, Carlsbad, CA, USA), using the following thermal cycling parameters: 95 °C for 10 min and 40 cycles of 95 °C for 15 s, 60 °C for 60 s. *Ywhag* and *β-actin* were used as internal controls respectively for oocyte and blastocyst samples. Relative mRNA level was calculated using the 2^−ΔΔCt^ method^52^. The primers were designed using Primer5 software (Supplementary Table S2).

### Statistical analysis

For all experiments, at least three biological replicates were conducted and the results are shown as the mean ± standard error of the mean (SEM). Statistical analyses were performed using SPSS19.0 software (SPSS Inc, Chicago, IL). Arcsin transformation was performed prior to statistical analysis of the percentage data. Difference between two groups was analyzed by independent-sample *t*-test, and multiple comparison tests were performed using one-way ANOVA followed by Duncan’s test^53^.

## Electronic supplementary material

Supplementary Information
