# Supplementary material for: Ascorbic acid induces global epigenetic reprogramming to promote meiotic maturation and developmental competence of porcine oocytes
Source: Sci Rep. 2018 Apr 17;8:6132. doi: 10.1038/s41598-018-24395-y (PMC5904140; doi:10.1038/s41598-018-24395-y)
Supplement: Supplementary file 1 — Supplementary Information [file 41598_2018_24395_MOESM1_ESM.pdf]

Ascorbic acid induces global epigenetic reprogramming to promote meiotic maturation and developmental competence of porcine oocytes

Xiao-Xia Yu, Yun-Hua Liu, Xiao-Man Liu, Pei-Chao Wang, Shuai Liu, Jia-Kun Miao, Zhi-Qiang Du*, Cai-Xia Yang*

Supplementary Information

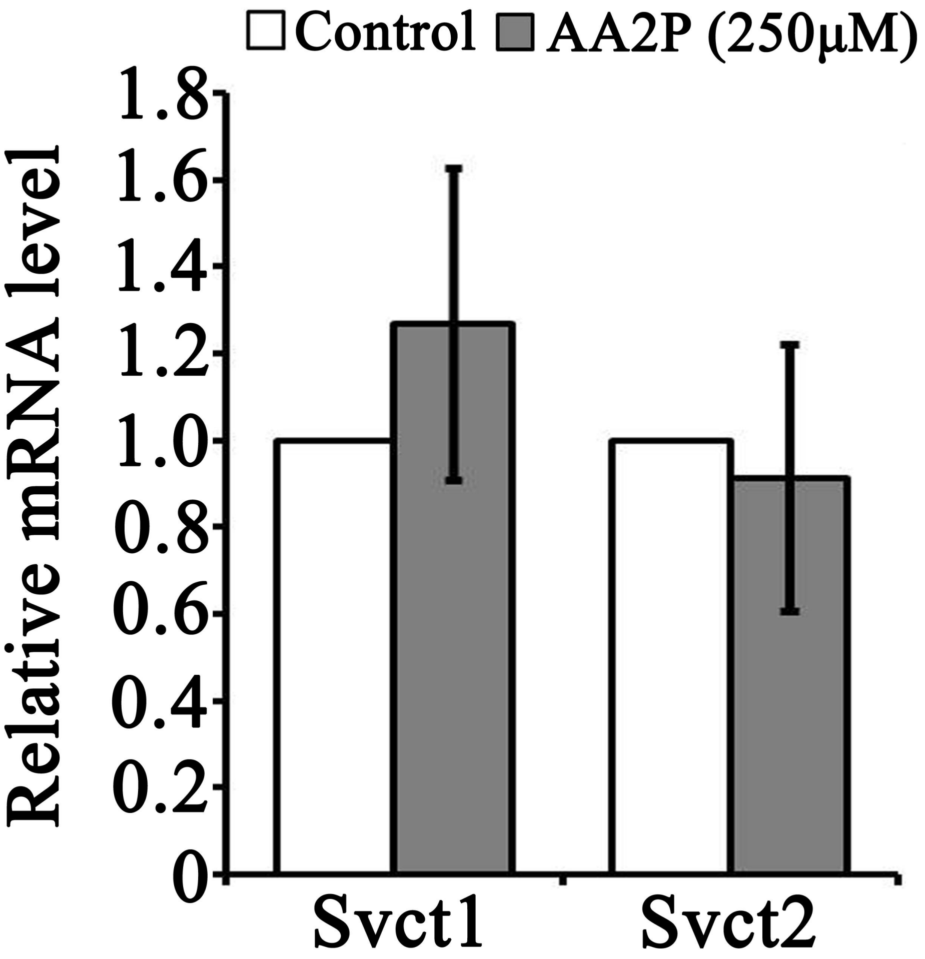

Figure S1. Transcript levels of sodium-dependent vitamin C transporters 1 and 2 (*Svct1* and *Svct2*) in porcine mature oocytes.

Table S1. Original RT-qPCR Ct values of Ywhag reference gene, Svct1 and Svct2 in pig MII oocytes.

Groups	Ywhag	Svct1	Svct2
Control	20.73±1.28	27.75±1.06	23.49±0.95
250μM AA2P	22.44±0.85	29.38±1.06	25.45±0.94

Table S2. Primer sequences used for real-time PCR.

Genes	Gene Bank accession No.	Primer sequence(5'-3')	Tm (℃)	Product size(bp)
β-Actin	XM_003124280.3	F: AGATCAAGATCATCGCGCCT<br>R: ATGCAACTAACAGTCCGCCT	60	170
Alkbh5	XM_005659000.1	F: CCAGTTCAAGCCTATCCGGG<br>R: GCGCATCTAACCTTGTCTTCC	60	173
Bax	XM_003127290.4	F: GCCGAAATGTTTGCTGACG<br>R: GCAGCCGATCTCGAAGGA	60	157
Bcl2	XM_021099593.1	F: CGTCCCAGCTCCACATCACC<br>R:AGTGCCCCACCGAAGGAGAA	60	130
Bmp15	NM_001005155.2	F:TGTGGTTTATCGCCATCAACTT<br>R: CGTGACATCCATCTCCGTCC	60	160
Caspase3	NM_214131.1	F:TTTGCGTGCTTCTAAGCCAT<br>R: GGCAGGCCTGAATTATGAAA	60	147
Cat	NM_214301.2	F:GCCGCCTATTTGCCTATCCT<br>R: TCCCCAGAATAGCGGGTACA	60	226
Dnmt1	XM_005662450.2	F:GCGTCTTGCAGGCTGGTCAGTA<br>R:CTTCTTATCATCGACCACGACGCT	60	152
Dnmt3a	NM_001032355	F: ATCAGTACGACGATGACGGC<br>R: CACCAAGAGATCCACGCACT	60	125
Dnmt3b	NM_001348900.1	F: ACCTGTCATCCGACACCTCT<br>R: CTCGGCATGAACCCACGTTA	60	155
Eed	XM_013979231.1	F: TGTGACTATTCTTGGGCGATTT<br>R:TTGGCTTTATGAGGATCTTCTACTT	60	153
Ezh2	NM_001244309.1	F: TGCAACACCCAATACTTACAAGC<br>R: ACTCTTTTGCTCCCTCCAAGT	60	101
Fto	NM_001112692.1	F: ATAGCAGCAGCATGAAGCGA<br>R: CAGCTGCCACTGCTGATAGA	60	143
G9a	NM_001101823.1	F: GGAGGAGCTGGGGTTTGAC<br>R:CAGAGGTGGCTGCTGAGTTG	60	254
Gdf9	NM_001001909.1	F: ACTCCAGAGCTTTGCGCTA<br>R: CCTGATGGAAGGGTTCCTGTC	60	169
Hif-1α	NM_001123124.1	F: GAAGATGAAATGAAGGCACA<br>R: TTCACAAATCAGCACCAAGC	60	235
Hif-2α	NM_001097420.1	F: ACCGTCAACCTCAAGTCAGC<br>R: TTCTGTCGTCGCAGTAGGTG	60	238
Kdm5b	XM_021064567.1	F: GTTTCCAAGAGCCTACCAC<br>R: GAACGGTTGATGCCACGAC	60	212
Kdm6a	XM_003360275.3	F: GCAGGCTCAGTTGTGTAACC<br>R: GGTTTACATGCCTGCTGTGC	60	150
Kdm6b	XM_013990021.1	F: GTGGAACCTGAACACCTGGG<br>R: TGGTGTGCCCTGTCTCATTG	60	150

Mettl3	XM_003128580.3	F: ACACTGCTTGGTTGGTGTCA<br>R: AATCTTTCGAGTGCCAGGGG	60	151
Mettl14	XM_003129231.4	F: GGATGTAGGTTTGGCCGACA<br>R: GGGGGTTCCAGAAGAATCACA	60	177
Mll2	XM_021091593.1	F: TGGAAGTGCAAGTGGTGTGT<br>R: GTACGGGGCGTGACAGATAG	60	138
Nanog	NM_001129971.1	F:AGGACAGCCCTGATTCTTCCACAA<br>R: AAAGTTCTTGCATCTGCTGGAGGC	60	156
Nsd1	XM_003123667.6	F: AGCCTTAAAGACAGAGCGCA<br>R: GGAGCCATTCTCTGAACCTCT	60	106
Oct4	NM_001113060.1	F: GGGGGTTCTCTTTGGGAAGG<br>R: TGTTGTCAGCTTCCTCCACC	60	128
Prdx2	NM_001244474.1	F: AGCATAAAAGGCTCCCCGTG<br>R: ATGACTGAAAGCTGCGTGGG	60	105
Prdx6	NM_214408.1	F: AGACCCAGCAGAAAAGGACG<br>R: CGCTGTCTCCATTCTTCCAGT	60	209
Setd2	XM_005669478.1	F: TAGCTCGCAAGCTGACTCAT<br>R: ACAGCCCCAAACTTCTGCAT	60	139
Sod1	NM_001190422.1	F: AAGAGAGGCACGTTGGAGAC<br>R: TTACACCACAGGCCAAACGA	60	216
Sod2	NM_214127.2	F: ACCCAAAGGGGAATTGCTGG<br>R: GAACAAGCGGCAATCTGCAA	60	159
Sox2	NM_001123197.1	F: CCCGTGGTTACCTCTTCTTCC<br>R: CGTTGATGGCCGTGCC	60	172
Suz12	XM_013981159.1	F: TCAGGATATTCATCGCCAACC<br>R: CTTCGGATTCAAGAAATTCAGACA	60	134
Suv39h2	NM_001039747.1	F: GGCAGGACGAACTCAACAGA<br>R: CTGATTCCCGGAGCTGGTTT	60	127
Svct1	XM_021084869.1	F: TTATCGCAGGGCTGTTAGGC<br>R: GGATCGGGTCAGGGATTGAG	60	180
Svct2	NM_214178.1	F: GAAATGGAAGAAGGGCGTAG<br>R: AGGTTGGGCTGATGGGTAAG	60	128
Tet1	NM_001315772.1	F: TACTCACCGACCCCATCCAT<br>R: AGCTGCATTAGCACCACCAT	60	198
Tet2	XM_013978993.1	F: GCTCATGCCCACAGAGACTT<br>R: CAGTACCTGAATGGCACCGT	60	207
Tet3	XM_005662450.2	F: TCTTCCGTCGTTCAGCTACTACAG<br>R: GTGGAGGTCTGGCTTCTTCTCAAA	60	127
Wtap	NM_001244241.1	F: GCGCCTTCCATTCGTCTTTC<br>R: GCCTCACTCAGTCGAACCTTT	60	136
Ywhag	XM_003124396.4	F:GGCCATGAAGAACGTGACAG<br>R: CATCCTGACATACGGCCTCC	60	224
